# Diving into the transcriptional landscape of leukemic stem cells in acute myeloid leukemia at single-cell resolution

**DOI:** 10.1007/s00432-026-06500-1

**Published:** 2026-05-29

**Authors:** Zuzanna Rzetelska, Lidia Gil, Francesco Buccisano, Dominik Dytfeld

**Affiliations:** 1 Department of Hematology, Transplantation and Cellular Therapy, Poznan University of Medical Sciences, Poznan, Poland; 2 Department of Biomedicine and Prevention, Tor Vergata University, Rome, Italy

**Keywords:** Acute myeloid leukemia, Leukemic stem cells, Single-cell RNA sequencing

## Abstract

**Purpose:**

The aim of this review is to summarize transcriptomic studies using single-cell RNA sequencing (scRNA-seq) and bulk RNA-seq to investigate leukemic stem cells (LSCs) population in acute myeloid leukemia (AML).

**Methods:**

Literature for inclusion was identified through a PubMed search. Twenty-seven studies were analyzed, covering various aspects of LSCs biology, including epigenetic, transcriptional, and translational gene regulation, metabolic reprogramming or cellular differentiation.

**Results:**

The reviewed studies highlight the complexity of LSCs, including their quiescent state, state-to-state cellular plasticity, and metabolic reprogramming from glycolysis to lipogenesis. Comparative analyses of matched diagnostic and post-treatment samples provide insights into the dynamic behavior of LSCs during therapy. The findings also identify potential therapeutic targets and strategies to selectively eradicate LSCs.

**Conclusion:**

Single-cell RNA sequencing technologies offer unprecedented resolution for studying LSCs in AML, revealing cellular heterogeneity and dynamic evolution within these critical populations. Despite these advances, standardized criteria for LSCs identification are still needed, and longitudinal studies at clinically relevant time points are essential to fully understand their role in disease progression and therapy resistance. Such approaches may ultimately uncover novel therapeutic vulnerabilities and improve clinical management of AML.

**Supplementary Information:**

The online version contains supplementary material available at 10.1007/s00432-026-06500-1.

## Acute myeloid leukemia

Acute myeloid leukemia (AML) is the most common acute leukemia in adults, characterized by the proliferation of abnormal, undifferentiated cells that impair normal hematopoiesis. AML patients suffer from pancytopenia leading to recurrent, life-threatening infections, anemia, and bleeding complications. Disease heterogeneity is well documented and patients are stratified according to their cytogenetic, molecular, and immunophenotypic profile. For fit patients, the standard treatment backbone remains a cytarabine and anthracycline based regimen, which yields a long-term survival of up to 40% in younger cohorts. In patients ineligible for intensive chemotherapy, the combination of azacitidine (AZA)/decitabina (DEC) and venetoclax (VEN) achieves a median overall survival (OS) of 14.7 months (DiNardo et al. 2020; Okoniewski et al. 2025). Relapse, which eventually occurs in over half of the patients who initially achieve complete remission (CR), represents the primary cause of treatment failure (Li et al. 2023).

## Introduction to leukemic stem cells

Numerous studies in solid tumor research have shown that the disease originates from cancer stem cells (CSCs), which reside at the apex of a cellular hierarchy (Bordenave et al. 2024; Kevat et al. 2025). This subpopulation can sustain long-term tumor growth and drive disease relapse, whereas most of the tumor comprises rapidly proliferating cells with limited self-renewal potential (Velten et al. 2021). This hypothesis has gained significant attention in hematologic malignancies, including AML. In 1994, Lapidot and colleagues first introduced the concept of leukemic stem cells (LSCs), also called leukemia-initiating cells (LICs), referring to AML-initiating cells capable of inducing full-blown leukemia following transplantation into severe combined immunodeficient (SCID) mice (Duy et al. 2021). The LSCs model arose from observations that only a subfraction of AML cells exhibiting an immature immunophenotype (CD34⁺CD38⁻) could re-establish leukemia (Bonnet and Dick 1997). Later studies demonstrated that LSCs can also be found within CD34⁺CD38⁺, and CD34⁻ subsets (Zeijlemaker et al. 2019; Arnone et al. 2020). However, the CD34⁺CD38⁻ compartment is considered as the most chemoresistant (Terwijn et al. 2014). LSCs share key immunophenotypic and metabolic characteristics with normal hematopoietic stem and progenitor cells (HSPCs), including self-renewal, quiescence, resistance to apoptosis, and increased drug efflux (Barreto et al. 2022). These similarities contribute to the intrinsic resistance of LSCs to conventional chemotherapy but also make difficult to differentiate them from normal HSPCs (Wu et al. 2023b). The distinction between HSPCs and LSCs is further complicated by the phenomenon of clonal hematopoiesis of indeterminate potential (CHIP), characterized by the accumulation of pre-leukemic mutations in HSPCs, a condition observed in approximately 10–20% of healthy individuals over the age of 70. These pre-leukemic stem cells (pre-LSCs) retain the capacity to produce normal blood and immune cells. While pre-LSCs often harbor CHIP-associated mutations (e.g., DNMT3A), some studies suggest that acquisition of additional, more proliferative mutations in later subclones may contribute to leukemic progression (Velten et al. 2021); however, this pattern is not universal, and evolution can also occur in the absence of clearly identifiable secondary hits (Young et al. 2019). Defining the LSCs population is also challenging due to significant inter-sample heterogeneity, which exists not only across AML subtypes classified by morphology but also within cases harboring the same genetic drivers (Zhou and Chng 2024). Furthermore, large intra-patient variability complicates characterization, and it may explain why some patients with genetic alterations associated with a favorable prognosis do not experience positive outcomes (Song et al. 2021; Li et al. 2023; Ortiz Rojas et al. 2024). Primarily, flow cytometry analyses demonstrated correlations between LSCs frequency and clinical outcomes such as lower CR rates, shorter relapse-free survival (RFS), and shorter OS in AML patients, as well as following allogeneic hematopoietic stem cell transplantation (allo-HSCT) (van Rhenen et al. 2005; Witte et al. 2011; Hwang et al. 2012; Terwijn et al. 2014; Plesa et al. 2015; Zeijlemaker et al. 2015, 2019; Jentzsch et al. 2017; Zahran et al. 2018; P et al. 2021). However, some studies failed to confirm the adverse prognostic impact of LSCs (Ding et al. 2012; Horibata et al. 2019; Boyd et al. 2023). Some data show that chemotherapy exerts similar cytotoxic effects on both LSCs and non-LSCs (Song et al. 2021). At the same time, others suggest that current chemotherapeutic agents, including cytarabine and anthracyclines, primarily target hyperproliferative blasts but spare LSCs (Ruvolo et al. 2019; Velten et al. 2021). Addressing the role of LSCs in resistance mechanisms in AML required the implementation of highly sensitive methods. In this review, we summarize studies published that have employed scRNA-seq and bulk RNA-seq to investigate the biology of LSCs in AML.

### From the beginning to RNA sequencing

Advances in technology and molecular biology techniques have enhanced our understanding of the genetic and transcriptomic landscape of AML. A revolution has emerged with the development of next-generation sequencing (NGS), which includes whole-genome sequencing (WGS), whole-exome sequencing (WES), and microarray (Ahmed and Zhong 2024). Furthermore, significant progress driven by the development of RNA-sequencing (RNA-seq) has led to the rise of a new field: transcriptomic—the study of the complete set of RNA transcripts expressed by a genome that includes messenger RNA (mRNA), long noncoding RNA (lncRNA), and microRNA (miRNA). RNA-seq enables comprehensive analysis of gene expression profiling. However, it only captures the average expression levels in cells, failing to reveal the specific characteristics of individual cell subpopulations (Li et al. 2023). To overcome this limitation, cell populations were analysed after sorting (Zhang et al. 2023). The transcriptomic study demonstrated that patients with a higher number of AML-related mutations had a higher transcriptional intratumor heterogeneity (ITH) score. AML patients with low cytogenetic or molecular risk had a low ITH, while patients with intermediate or high cytogenetic or molecular risk showed a higher ITH score, which was associated with poor OS and higher relapse rates (Li et al. 2023).

### LSCs scores

The ELN guidelines stratify patients into favorable, intermediate, and adverse prognostic risk groups (Döhner et al. 2022). Despite the continuous improvement of classification systems, genetic-molecular landscape at diagnosis still may fail at predicting prognosis on an individual basis especially in the intermediate-risk group and in patients not treated by intensive chemotherapy. Therefore, based on transcriptomic studies of LSCs, attempts have been made to develop a prognostic model using LSCs-related genes that would better stratify patients in terms of risk. The first LSCs score, known as LSC52, was published in 2010 and was based on the expression of 52 genes identified using microarray technology. High expression of an LSCs gene signature was independently associated with adverse outcomes in AML patients (Gentles et al. 2010). Using bulk RNA-seq, the most commonly referenced LSC-17 score (also known as LSC-Ng) was invented, which takes into account the expression of the following genes: *DNMT3B*, *ZBTB46*, *ARHGAP22*, *LAPTM4B*, *MN1*, *CPXM1*, *SOCS2*, *SMIM24*, *NGFRAP1*, *EMP1*, *AKR1C3*, *CDK6*, *GPR56*, *KIAA0125*, *PRAM1*,* SPINK2*, and *HOPX* (Ng et al. 2016; Bill et al. 2020). Additionally, in the pediatric population, the LSC-6 score was developed, which includes the expression of *DNMT3B*, *GPR56*, *CD34*, *SOCS2*, *SPINK2*, and *FAM30A* (Elsayed et al. 2020). EPPERT-LSC (also known as LSC-up or LSC-R), consists of the expression levels of 44 LSCs-associated genes (Eppert et al. 2011). Transcriptome analysis of AML cohorts (BeatAML, TCGA, NTUH, AMLCG1999, HOVON) led to the development of a four-gene prognostic index (4-PI), comprising *CYP2E1*, *DHCR7*,* SQLE*,* IL2RA*, which stratifies patients into risk groups regardless of ELN classification (Ortiz Rojas et al. 2024). Patients with high 4-PI scores had lower survival rates compared to those with low 4-PI scores and exhibited a greater abundance of LSCs and high LSC-17 and EPPERT-LSC scores. 4-PI can be used as a refining marker of the ELN classification, mainly in intermediate-risk patients (Ortiz Rojas et al. 2024). The other idea was to use the expression of the lncRNA score system, which was correlated with LSCs signatures (Tsai et al. 2019). To date, no gene expression signature has been incorporated into risk classification systems, mainly due to limited validation, poor reproducibility, and the complexity of utilizing large gene panels in clinical practice.

### Single-cell RNA-sequencing

**Fig. 1:**
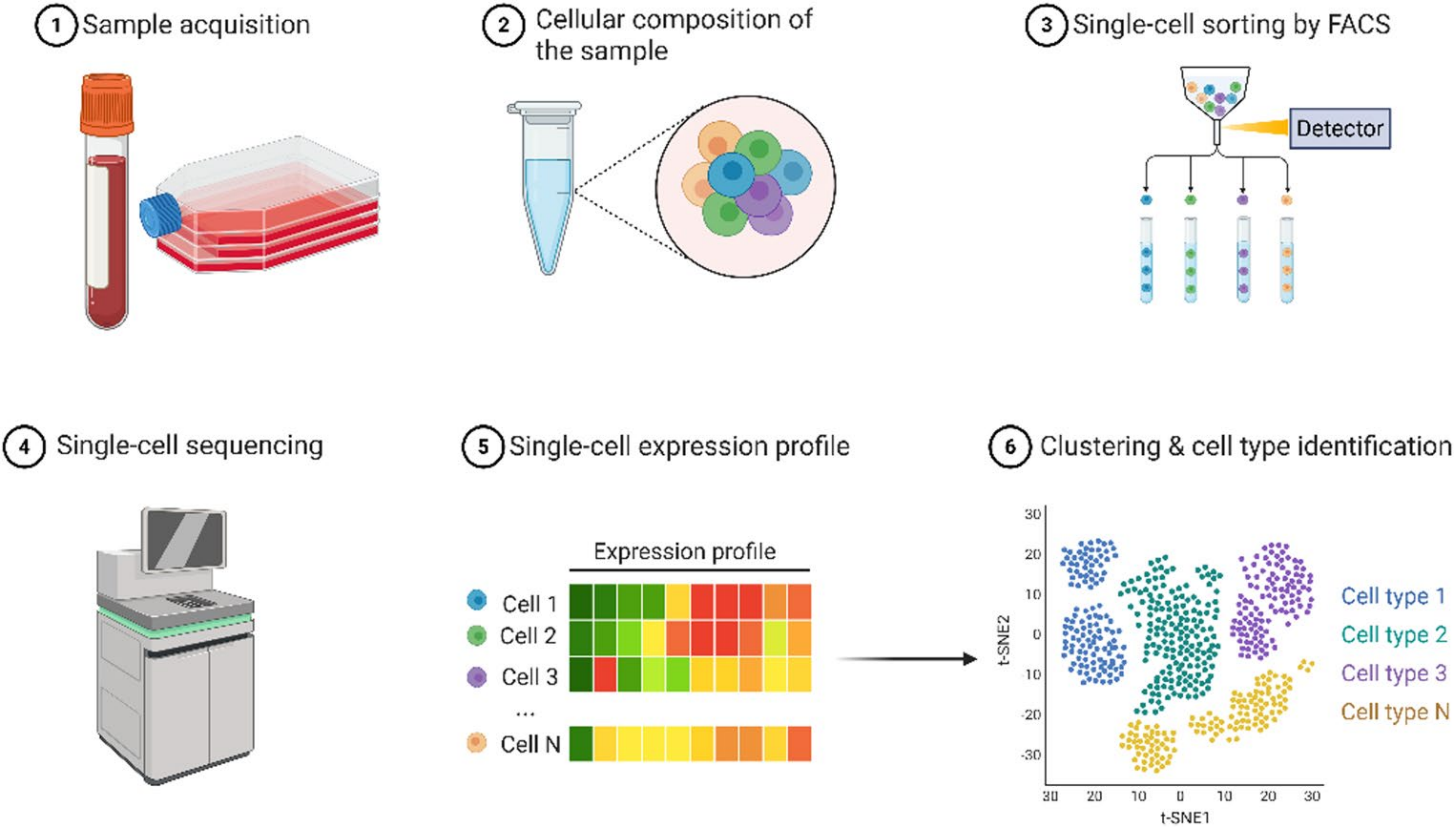
Diagram illustrating the workflow and concept of the single-cell technique. Created in BioRender. Rzetelska (2026) https://BioRender.com/2t2stkb

Bulk genomic profiling has advanced cancer research but lacks the resolution to capture intratumoral heterogeneity, particularly in rare subsets such as LSCs in AML (Yan et al. 2017; Song et al. 2021). The first transcriptomic analysis of AML at single-cell resolution was published in 2017 by Hughes et al., based on a single patient sample. This was followed by the high-impact study by van Galen et al. in 2019, which systematically profiled thousands of cells from multiple AML patients, defining transcriptional hierarchies of leukemic subpopulations (Yan et al. 2017; van Galen et al. 2019a). Single-cell RNA-seq (scRNA-seq) has revolutionized our understanding of cellular heterogeneity in tissues (Fig. 1). By profiling gene expression at the level of individual cells, scRNA-seq can uncover cellular diversity, including rare cell populations. Other significant advancements include the use of artificial intelligence, deep learning technology, and data accessibility through open-access genomic databases. Several scRNA-seq platforms are available on the market, differing in protocol complexity, costs, the number of output cells, sequencing depth, and whether they provide full or partial transcripts coverage (Caprioli et al. 2021).

## Methods

Literature for inclusion was identified through a PubMed search in January 2024, combining AML terms with LSCs terms and scRNA-seq terms. The full list of search terms is shown in Online Resource 1. Two filters were applied to the PubMed search: published in the last 10 years and written in English. A flow diagram summarizing the study selection process is shown in Online Resource 2. Table 1 provides a comparative overview of the key studies included in this analysis.

**Table 1:** A comparative summary of the included studies

Year & References	Population/cell lines	Material	Number of analysed cells	Assessment time	Data avaibility	Key finding
Bakhtiari et al. (2024)	Pediatric AML patients	20 patients BM samples	No data	Pretreatment, post-treatment, and at relapse	GEO database GSE235923	ARMH1 identified as a novel biomarker for disease progression and relapse in pediatric AML, correlated with LSC6 score.
Chen et al. (2024)	Adult AML patients	20 patients BM samples	41,945 cells	Pretreatment	GEO database GSE240618 Single Cell Portal SCP2316	MPO and TRH expression levels within LSCs could serve as biomarkers for predicting responses.
Sjövall et al. (2024)	Murine MLL-AF9 leukemia	BM cells harvested from leukemia-engrafted mice	18,426 cells	After engraftment	ArrayExpress E-MTAB-13,567	LSCs adaptation to defective ribosome assembly is associated with an increase in ribosome biogenesis and deregulation of the transcription factor landscape.
Zeng et al. (2024)	Adult AML patients, pediatric AML patients, 18 MPAL patients	318 BM patient samples	1,223,411 cells	Pretreatment, after engraftment	Available from the corresponding authors on request	Distinct AML hierarchies sustained by distinct LSC populations can co-exist within a subset of patients.
Tian et al. (2024)	R/R adult AML patient vs. healthy controls	8 BM patient samples and 4 healthy controls BM	27,754 cells vs. 24,325 cells	Before subsequent treatment	GEO databases: GSE223844 and GSE116256	The significant role of *ENO1* in the proliferation, differentiation, and treatment resistance of LSCs.
Velasco-Hernandez et al. (2024)	Pediatric/young adult AML patients	11 BM patient samples	119,000 cells	Pretreatment, at relapse	European Genome-Phenome Archive EGAS00001005980	The HIF/hypoxia pathway signature is attenuated in LSCs compared with more differentiated AML cells, but is more expressed than in healthy hematopoietic cells.
Bordeleau et al. (2024)	Adult AML patients	20 BM patient samples	103,690 cells	Pretreatment	GEO database GSE241989	Study uncovered 15 candidate LSC markers providing potential therapeutic strategies.
Zhang et al. (2023)	Pediatric AML patients	26 BM patients samples	227,842 cells	Pretreatment and posttreatment	Beijing Institute of Genomics, Chinese Academy of Sciences and China National Center for Bioinformation HRA001009, HRA000996, HRA001021	LSCs and OXPHOS as two major chemoresistant features in human AML patients. *CD69* may serve as a potential biomarker in defining a subpopulation of chemoresistant LSCs.
Beneyto-Calabuig et al. (2023)	AML patients	19 BM patients samples	88,602 cells	Pretreatment, posttreatment, and at relapse	European Genome-Phenome Archive EGAS00001007078	CloneTracer reveals a differentiation landscape that mimics its healthy counterpart and may determine biology and therapy response in AML.
Wu et al. (2023a)	C57/BL6 murine AML model	BM cells were harvested from leukemia-engrafted mice	41,078 cells	Pretransplanted, preleukemic BM cells at 2 weeks, 4 weeks and 8 weeks after engraftment	GEO database GSE142645	GMP-like cells (preleukemic cells) did not gain a significantly increased AML or LSCs signature compared to HSPCs.
Naldini et al. (2023)	AML adult patients, murine model	19 BM and 1 PB patients samples, 16 healthy donors	142,613 cells vs. 130,288 cells	Pretreatment, posttreatment, after engraftment	GEO database GSE185993	MiR-126 (high) LSCs are enriched at diagnosis in chemotherapy-refractory AML and at relapse and its presence stratifies patients for survival in large AML cohorts Evidence for a “classical” LSC model in NPM1mut AML, where non-response/relapse was strongly correlated to a high proportion of quiescent miR-126high LSC already present at diagnosis OxPhos (low) miR-126 (high) cells display enforced stemness and quiescence features.
Wu et al. (2023b)	Adult AML patients	5 NK (normal karyotype)-AML (M4/M5) patients and 1 healthy donor	36,865 cells vs. 2,423 cells	Pretreatment	Available from the corresponding author on request	Identification of a distinct LSCs-like cells population with possible biomarkers in AML with a normal chromosomal karyotype. Potential biomarkers and prognostic predictors for clinical applications in NK-AML.
Li et al. (2023)	R/R adult AML patients	7 R/R BM patients samples Matched BM samples from 6 patients	44,151 cells vs. 74,414 cells	Pretreatment and posttreatment	Beijing Institute of Genomics, Chinese Academy of Sciences and China National Center for Bioinformation HRA001240	Compared to proliferating stem/progenitor-like cells (PSPs), a subpopulation of quiescent stem-like cells (QSCs) were involved in the chemoresistance and poor outcomes of AML. Longitudinal scRNA-seq analyses demonstrated that PSPs were reprogrammed to obtain a QSC-like expression pattern during chemotherapy in refractory AML patients.
Lambo et al. (2023)	Pediatric AML patients	28 BM or PB patients samples	684,031 cells	Pretreatment and posttreatment, at relapse	GEO database GSE235063	Cellular hierarchies transitioned toward a more primitive state regardless of subtype upon relapse. Transcriptional networks shift from myeloid toward lymphoid programs upon relapse.
Jia et al. (2022)	Adult AML patients	6 BM patients samples	60,402 cells	Pretreatment	Data was not provided	A cell population with *CRIP1*high, *LGALS1*high, and *S100A*high features, showing characteristics of granulocyte-monocyte progenitors, was associated with a poor prognosis in AML. Cell populations marked by CD34^+^CD52^+^ or CD34^+^CD74^+^DAP12^+^ were related to good response to induction therapy.
2022 (Zhai et al. 2022)	Adult AML patients	6 BM patients samples	5,612 cells	Pretreatment and posttreatment	European Genome-Phenome Archive EGAD00001008373	Gene expression varies widely between patients and diagnosis-relapse pairs, even with shared initiating events. Differences reflect clonal evolution and are most pronounced in cases with large copy number changes at relapse.
Thakral et al. (2023)	Pediatric AML patients	3 BM patients samples	40,000 cells	Pretreatment	Available from the corresponding author on request	LSC demonstrates intra-patient heterogeneity in addition to inter-patient diversity. The dormancy and quiescence genes are overexpressed in LSC similar to HSC but display considerable heterogeneity with multiple LSC clusters showing variable level of expression.
Stetson et al. (2021)	Adult AML patients	5 BM patients samples	721 cells	Pretreatment and posttreatment	Data was not provided	Despite the presence of vast transcriptional heterogeneity at the single cell level, pathway analysis identified common signaling networks involving metabolism, apoptosis and chemokine signaling that evolved during AML progression and become a signature of relapse samples.
Niu et al. (2021)	Adult AML patients	3 BM patients samples	3,300 cells per treatment and per patient	Cells treated with DMSO, SLC-391, or venetoclax, alone or in combination for 16 h	European Genome-Phenome Archive EGAS00001004663	AXL inhibition, alone or in combination with venetoclax, potentially targets intrinsic metabolic vulnerabilities of AML stem/progenitor cells and inhibits OXPHOS SLC-391 exhibits synergistic cytotoxicity with venetoclax in killing AML cells.
Duy et al. (2021)	Adult AML patients	BM or PB patients- derived culture samples	4,627 cells	Pretreatment and posttreatment	GEO database GSE146544	Data argue against selective enrichment for LSCs immediately following Ara-C treatment and suggest that survival post-chemotherapy may be linked instead to stochastic induction of senescence-like programs in cells independent of whether they correspond to LSC or bulk AML populations.
Velten et al. (2021)	Adult AML patients	4 BM patients samples	618 to 1430 cells per patient	Pretreatment	European Genome-Phenome Archive EGAS00001003414	LSCs, HSCs, and pre-leukemic stem cells can be identified and molecularly profiled by combining single-cell transcriptomics with lineage tracing using both nuclear and mitochondrial somatic variants.
Wesely et al. (2020)	AML-4.10 iPSC (induced pluripotent stem cells) cell line	separated iLSCs	Not indicated	Analysis on day 16, day 23 and day 30	GEO database GSE124992	iLSCs establish a hierarchy that resembles that of normal HSCs by giving rise to heterogeneous populations corresponding to immature and more mature progenitor cells of diverse hematopoietic lineages.
Sachs et al. (2020)	murine Mll-AF9/NRAS^G12V^ leukemia model	BM cells were harvested from leukemia-engrafted mice	Not indicated	18 days after transplant	GEO database GSE140896	Self-renewal and proliferation are mutually exclusive features of LSCs and suggest that targeting self-renewal, in addition to proliferation, may be an important strategy to prevent AML relapse.
Domingues et al. (2020)	*Kat2a*-WT and *Kat2a*-KO MLL-AF9 murine leukemia models	BM cells were harvested from leukemia-engrafted mice	7360 cells	*Kat2a*-wildtype vs. *Kat2a*-knockout	GEO database GSE118769	*Kat2a* contributes to leukemia propagation through preservation of leukemia stem-like cells.
Izaguirre-Carbonell et al. (2019)	MLL-AF9 murine leukemia models	BM cells were harvested from leukemia-engrafted mice	~ 1000 cells	Cells harboring sgRNAs against Renilla domain vs. against JmjC domain	Data was not provided	JMJD1C, a histone demethylase, was essential for AML LSC self-renewal, as loss of *JMJD1C* activity led to its depletion.
van Galen et al. (2019a)	Adult AML patients	35 BM patients samples	30,712 vs. 7,698 cells	At diagnosis and during treatment	GEO database GSE116256	A combination of transcriptomics and mutational analyses in single cells from AML reveals the existence of distinct functional subsets and their associated drivers.
Pollyea et al. (2018)	Adult AML patients	33 BM patients samples	Not indicated	At baseline and after two and four days of therapy	GEO database GSE116481	LSC compartment can be effectively eradicated by targeting LSC-specific metabolic properties. The population eradicated by venetoclax + azacitidine strongly expressed molecular signatures identified in primary human LSCs.

### Immunophenotype defined by scRNA-seq

Early investigations into LSCs focused on identifying a unique immunophenotype that distinguishes them from healthy HSPCs. Some lineage markers are always negative on CD34^+^CD38^−^ HSPCs such as CD2, CD7, CD11b, CD19, CD22, CD56, CLL-1 (known also as CD371, CLEC12A) (Hauswirth et al. 2007; Hosen et al. 2007; van Rhenen et al. 2007; Herrmann et al. 2020). ScRNA-seq studies have also focused on determining the unique profiles of genes encoding LSCs surface antigens (van Galen et al. 2019a; Velten et al. 2021; Wu et al. 2023b; Velasco-Hernandez et al. 2024; Zhou and Chng 2024; Bordeleau et al. 2024). Most LSCs express high *CD34* levels, including AML with inv(16) and t(8;21) (Velasco-Hernandez et al. 2024). By contrast, MLLr (*KMT2A* rearrangement) and *NPM1*-mutated patient-derived samples mainly consisted of *CD34* negative cells (von Palffy et al. 2023; Velasco-Hernandez et al. 2024). Table 2 presents the repertoire of surface antigens expressed by LSCs, as determined by single-cell RNA sequencing. *NPM1-*mutated AML blasts enriched for LSCs activity highly expressed *CD33*, *CD123*, *CD117*, *CD244/NKR2B4*, *CD200*, *C3AR*, and *GPR56* the adhesion G protein-coupled receptor G1. GPR56 was identified as a cell surface marker of LSCs on both CD34^+^ and CD34^−^ cells (Velten et al. 2021). *C3AR*^+^*GPR56*^+^ cells but not *C3AR*^+^*GPR56*^−^ cells from a patient with primary *NPM1*-mutated AML engrafted (von Palffy et al. 2023). Some surface LSCs subsets correlated with good response to induction chemotherapy like *CD34*^+^*CD52*^+^ and *CD34*^+^*CD74*^+^*DAP12*^*+*^ (Jia et al. 2022). On the other hand *CD9*, *CD82*, *CDK6*, *CD69* a type II transmembrane C-type lectin receptor, *CD79A*, *CD317/BST2*, *RGS10*, and *B2M* were upregulated in the refractory group (Zhang et al. 2023; Tian et al. 2024). With regard to the clinical application of scRNA analyses, it is important to remember that transcriptional levels of immune markers do not always correlate with their corresponding surface protein expression, as demonstrated by studies comparing mass cytometry and flow cytometry with scRNA-seq data. The relationship between mRNA and protein abundance is cell–type–dependent. Therefore, studies systematically assessing the concordance between transcriptional and protein levels are needed to enable the direct clinical interpretability of transcriptomic data (Su et al. 2024).

**Table 2:** A comprehensive overview of the expression of genes encoding surface antigens in leukemic stem cells, identified through single-cell RNA-sequencing

Surface markers genes	Details	References
*CD7*	Transmembrane glycoprotein expressed by 30% of AML, associated with more aggressive disease and resistance to standard therapy (Gomes-Silva et al. 2019)	Bordeleau et al. (2024)
*CD24*	Mucin-like highly glycosylated molecule studied as a CSCs marker in solid cancers (Yang et al. 2023b)	Bordeleau et al. (2024)
*CD33*	Immunoglobulin superfamily molecule highly expressed in the majority of *NPM1*-mutated LSCs (Krupka et al. 2014; Laing et al. 2017)	von Palffy et al. (2023)
*CD36*	Multiliganded receptor related to fatty acid uptake (Stetson et al. 2021; Guerrero-Rodríguez et al. 2022)	van Galen et al. (2019a), Zhou and Chng (2024)
*CD44*	Transmembrane glycoprotein responsible for adhesion, elevated expression in IDH-mutant AML cells (Lyu et al. 2025)	Stetson et al. (2021), Wu et al. (2023b)
*CD46*	Regulator of the classical and alternative complement activation cascade in the innate immune system (Wu et al. 2023b)	Zhou and Chng (2024)
*CD47*	Transmembrane protein activating “don’t eat me” signal resulting in inhibition of phagocytosis (Majeti et al. 2009; Yang et al. 2023a)	van Galen et al. (2019a), Wu et al. (2023b), Velasco-Hernandez et al. (2024), Zhou and Chng (2024)
*CD52*	Glycopeptide related with poor prognosis (Chen 2024)	Jia et al. (2022), Li et al. (2023), Velasco-Hernandez et al. (2024)
*CD56 (NCAM1)*	Highly overexpressed by *KMT2A* rearrangement cells, associated with resistance to cytarabine, daunorubicin, and midostaurin (Bordeleau et al. 2024)	Bordeleau et al. (2024)
*CD74*	Transmembrane protein responsible for cell survival in a network with LGALS3 (Ruvolo et al. 2019)	van Galen et al. (2019a), Jia et al. (2022), Naldini et al. (2023), Zhou and Chng (2024)
*CD82*	Tetraspanin family protein	Wu et al. (2023b), Velasco-Hernandez et al. (2024), Tian et al. (2024)
*CD90*	Membrane GPI-anchored protein with one Ig V-type superfamily domain plays a role in cell-cell and cell-matrix interactions (Sauzay et al. 2019)	Wesely et al. (2020)
*CD96* (*TACTILE*)	Co-stimulatory receptor belonging to the immunoglobulin superfamily associated with dismal survival (Zhou and Chng 2024)	Wesely et al. (2020), Bordeleau et al. (2024)
*CD99*	Highly O-glycosylated transmembrane protein plays a role in apoptosis, cell adhesion and leukocyte diapedesis (Kadam et al. 2024)	Wu et al. (2023b), Naldini et al. (2023), Velasco-Hernandez et al. (2024)
*CD123* (*IL3RA*)	Interleukin-3 (IL-3) receptor that activates the JAK/STAT, MAPK and PI3K pathways to promote myeloid differentiation (Dreyzin et al. 2025)	von Palffy et al. (2023)
*CD133* (PROM1)	Transmembrane glycoprotein playing role in CSCs survival	Wu et al. (2023b), Bordeleau et al. (2024)
*CD134 (TNFRSF4)*	Member 4 of tumor necrosis factor receptor (TNFR) superfamily (Naldini et al. 2023; Bordeleau et al. 2024)	Naldini et al. (2023), Bordeleau et al. (2024)
*IL1RAP*	Interleukin-1 (IL-1) receptor accessory protein plays role in amplification and transmission the signals of IL-1 family of cytokines, including IL-1, IL-33, IL-36 (Zhang et al. 2024)	(van Galen et al. 2019a; Zhou and Chng 2024)
*NPR3*	Atrial natriuretic peptide receptor	Bordeleau et al. (2024)
*SLC38A1 (SNAT1)*	Solute carrier family 38 member 1	Bordeleau et al. (2024)
*ADGRG1*	G protein-coupled receptor family that plays a key role in cell adhesion and cell-cell interactions (Singh and Lin 2021)	Velten et al. (2021), von Palffy et al. (2023), Bordeleau et al. (2024)
*ADRA2A*	Alpha-2 A adrenergic receptor	Bordeleau et al. (2024)
*CALCRL*	G protein-coupled neuropeptide receptor calcitonin receptor-like, a marker of stemness (Gluexam et al. 2019)	Bordeleau et al. (2024)
*CD109*	Glycosyl phosphatidylinositol-linked glycoprotein negatively regulating TGF-β signaling (Mii et al. 2019)	Bordeleau et al. (2024)
*MPIG6B*	Megakaryocyte and Platelet Inhibitory Receptor G6b, member of the immunoglobulin (Ig) superfamily	Bordeleau et al. (2024)
*TMIGGD2*	Transmembrane and immunoglobulin domain containing 2 required for the development and maintenance of AML and self-renewal of LSCs (Wang et al. 2024)	Bordeleau et al. (2024)
*PLEK*	Megakaryocyte marker	Wesely et al. (2020)
*PF4*	Megakaryocyte marker	Wesely et al. (2020)
*GP9*	Megakaryocyte marker	Wesely et al. (2020)
*CD41a*	Megakaryocyte marker	Wesely et al. (2020)

### Gene expression control at the transcriptional and translational levels

Transcriptional regulation represents a fundamental mechanism of gene expression control in LSCs. Ruinan et al. showed that resistant CD34^+^ AML cells exhibit uncontrolled transcription compared with sensitive cells. In study by Stetson et al. LSCs (CD34^+^/CD38^−^/CD90^−^/CD45RA^−^/Lin^−^/7-AAD^−^ cells sorted by flow cytometry ) clusters dominant at relapse exhibited low expression of *KLF6*. *KLF6* is a transcription factor (TF) that acts as a tumor suppressor, helping to prevent uncontrolled cell growth and tumor development. Additionally, other pro-apoptotic transcription factors, including *BCLAF1*, *HMGB2*, and *TP53* along with pro-apoptotic target genes such as *BAX*, *CASP1/2/6*, and *FAS* were also downregulated. In contrast, during disease progression, anti-apoptotic transcription factors *EP300* and *JUND*, as well as other anti-apoptotic genes, such as *BCL2A1*, *c-FLIP*, *MCL1*, and *SOCS3*, were upregulated (Stetson et al. 2021). The expression of a member of the core-binding factor family of transcription factors *RUNX1* was significantly higher in LSCs (adherent cells CD34^+^/CD38^−^/CD90^+^/CD45RA^−^/CD49f^+^ derived from AML-4.10 and AML-4.24 cell lines) and was associated with the 42-gene LSC signature (Wesely et al. 2020). Whereas, Chen et al. revealed that LSCs (the malignant cell cluster with the highest gene set variation analysis (GSVA) scores, calculated from the LSC-Ng, LSC-R, and LSC52 gene sets, was identified by the algorithm) from CR patients showed enrichment of *MYC*-driven transcriptional programs (MYC Targets V1 and V2), including pathways related to ribosome biogenesis, nucleotide metabolism, cell cycle progression, and protein synthesis, compared to LSCs from patients who do not achieve complete remission (non-CR) (Chen et al. 2024). Similar observations were made using RNA-seq, where reduced ribosomal biogenesis and protein synthesis were identified as potential mechanisms by which LSCs (GEO dataset GSE74246) acquire stress resistance. The LSCs population showed significant downregulation of structural proteins of the large ribosomal subunit (*RPL35* and *RPL36*), as well as ribosomal and splicing-related RNA-binding proteins (RBPs), such as *RPL7A*, *RPL11*, *RPL18*, and *RPS11*, which are involved in post-transcriptional gene regulation (PTGR) (Saha et al. 2019). These findings suggest that LSCs rely on altered ribosome dynamics to maintain a cancer-specific translatome. Velasco-Hernandez et al. likewise confirmed that LSCs (CD34⁺/CD38⁻ cells were clustered using an unsupervised algorithm, with the cluster exhibiting the highest LSC6 scores identified) in diagnostic samples exhibited high activation of the translation process (Velasco-Hernandez et al. 2024). Moreover, LSCs (c-Kit^+^) had higher ribosome biogenesis cell scores and represented one of the most highly translating cell types in the BM. *eIF6* acts as an anti-association factor, blocking 60 S and 40 S subunits from forming active 80 S ribosomes. *eIF6* overexpression resulted in a reduction in the frequency of c-Kit^+^ cells but did not prevent the disease from propagating in secondary hosts. LSCs with induced *elF6* overexpression displayed higher LSCs scores. Adaptation to defective ribosome assembly leads to the upregulation of genes associated with the LSCs’ identity, including *C/EBPε*, *GFI1*, and *IRF8* (Sjövall et al. 2024).

### Mi-RNA

Noncoding RNAs, such as microRNAs and lncRNAs, regulate gene expression post-transcriptionally and have been implicated in LSCs regulation based on bulk sequencing data (Bill et al. 2019). *EGFL7* (Epidermal Growth Factor-Like Domain 7) harbors the intronic *miR-126*, whose expression is elevated in LSCs-enriched AML subpopulations. MiR-126 maintains LSCs (leukemia-like cluster based on unsupervised clustering) in a quiescent state by inhibiting the PI3K/AKT signaling pathway, thereby promoting chemoresistance (Li et al. 2023). The *miR-126* high cells showed enrichment of LSC17 signatures and *ADGRG127*, an LSC-associated gene. In refractory patients high *mi*R-126 LSCs persist after induction chemotherapy (Naldini et al. 2023) what supports a classical LSC model in which treatment resistance is linked to the presence of cells already detectable at diagnosis.

### Maintaining quiescence status

Dormant cancer cells are in a reversible non-proliferative state and are closely associated with tumor recurrence (Niu et al. 2022). LSCs are believed to reside in the G0 phase of the cell cycle (Stelmach and Trumpp 2023). Numerous single-cell analyses have confirmed that LSCs are predominantly found in the G0 phase, exhibiting a quiescent phenotype (Wesely et al. 2020; Song et al. 2021; Stetson et al. 2021; Li et al. 2023; Naldini et al. 2023; Velasco-Hernandez et al. 2024). Chemoresistant patients showed a higher proportion of LSCs in the G0/G1 phase and a lower proportion of cells in the S and G2/M phases compared to those who achieve CR (Stetson et al. 2021; Naldini et al. 2023; Chen et al. 2024). The proportion of quiescent cells significantly increased at relapse (Li et al. 2023). LSCs (CD34⁺/CD38⁻/CD69 + HSC-like leukemia cells) from relapsed patients display decreased expression of genes related to mTOR and STAT3 pathways, along with downregulation of key cell cycle regulators such as *CDK6* and *CCND1* (Zhang et al. 2023). LSCs (defined above) showed enrichment of the Hedgehog signaling pathway, which is thought to play a critical role in maintaining LSCs quiescence (Chen et al. 2024). The CXCR4-mediated microenvironmental interaction molecules like *PIM1* were also found upregulated (Zhang et al. 2023). Other genes associated with cell cycle regulation have been identified, including *IKZF2*, which was recently reported to drive LSCs self-renewal and inhibit myeloid differentiation by disrupting the CEBPD/E-driven transcriptional regulation program (Li et al. 2023). Additional genes such as *MDM4*, *PTPRS*, *DNMT1*, and *GMNN* also contribute to the negative regulation of cell cycle entry (Wesely et al. 2020).

**Fig. 2:**
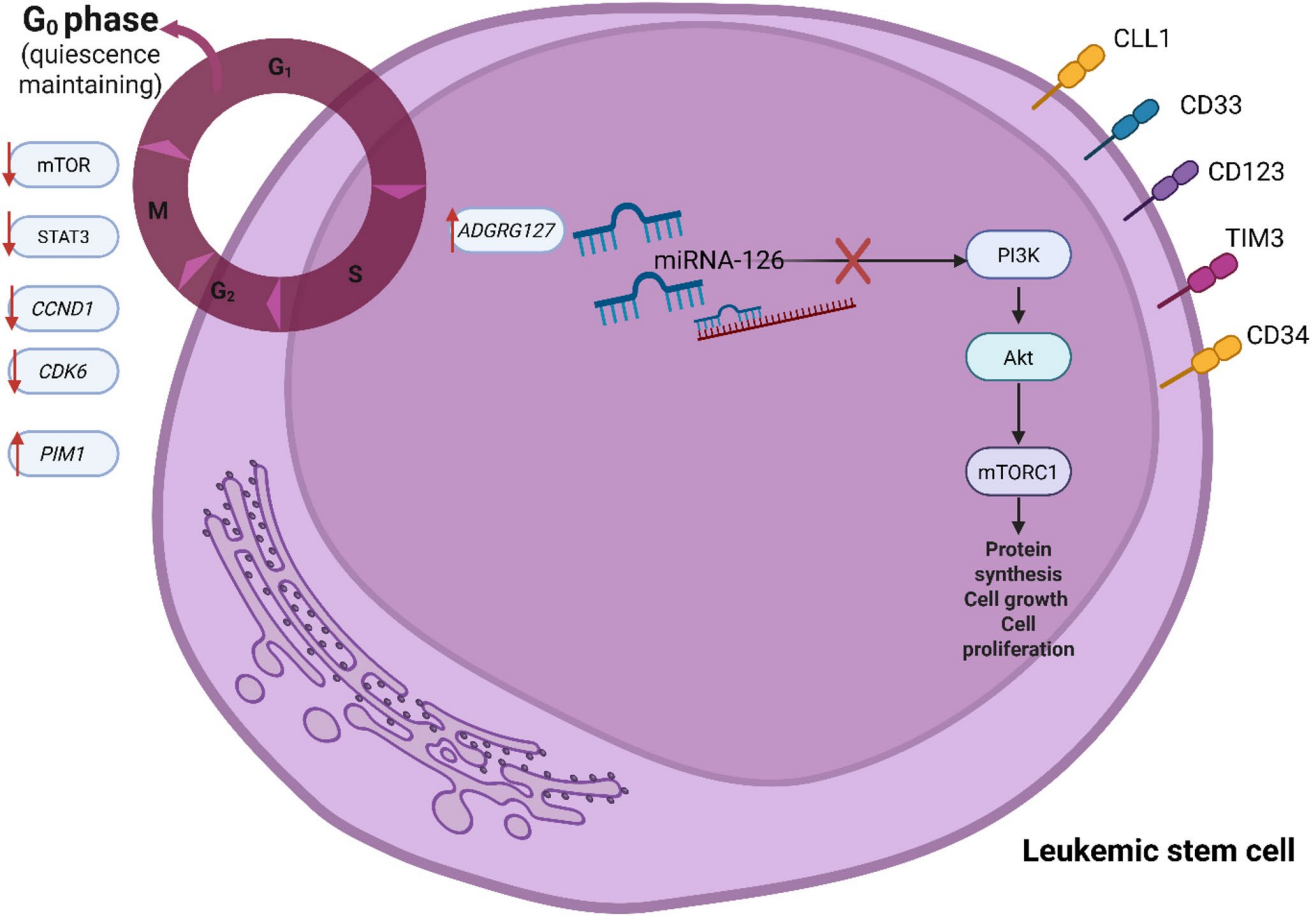
Maintenance of quiescence in leukemic stem cells and miRNA function in LSCs. Created in BioRender. Rzetelska (2026) https://BioRender.com/2t2stkb

Hypoxia represents a self-defense mechanism to maintain cells in a dormant state, desensitizing them to chemotherapy. Cells respond to hypoxia through hypoxia-inducible factors (HIFs), which trigger the expression of hypoxia-regulated genes engaged in cell proliferation and survival. Treatment-resistant AML cells were believed to preferentially reside in the hypoxic endosteal BM niche, which provides protection from the pro-apoptotic effects of chemotherapeutic agents. However, two scRNA studies have not confirmed this. Velasco-Hernandez et al. 2024 consistently found an inverse correlation between the hypoxia signature and cell stemness, manifested as a gradual enrichment of the hypoxia signature from LSC^34^ to differentiating non-LSC^34^ and non-LSC^38^ cells (Velasco-Hernandez et al. 2024). Additionally, analysis of a pediatric population revealed patient-specific heterogeneity in both LSC6 scores and hypoxia scores in paired diagnostic–relapsed samples (Lambo et al. 2023). A diagram illustrating maintenance of quiescence and miRNA function in LSCs is shown in Fig. 2.

### State-to-state cellular plasticity

Understanding how LSCs regulate self-renewal, maintain quiescence, or enter senescence is essential for revealing their role in leukemia progression and therapy resistance. Many genes expressed in quiescent cells are also senescence-associated genes, potentially indicating an overlap between senescence and dormancy (Duy et al. 2021). The classical model, in which chemotherapy-resistant LSCs accumulate over successive treatment cycles, has recently been challenged due to emerging evidence of heterogeneous and transient senescent states within this population. Cellular senescence is a stress-induced state characterized by growth arrest and aberrant metabolism. ScRNA-seq analysis showed that senescent cells could repopulate AML. However, the senescence-like AML cells collected three days after exposure to Ara-C at nadir showed no enrichment of LSCs markers that could explain the association with increased engraftment (Duy et al. 2021). Genes linked to SASP (Senescence-Associated Secretory Phenotype), a set of molecules secreted by senescent cells (e.g., cathepsins and IL8), were upregulated upon Ara-C treatment. Most studies analyzed samples at diagnosis and relapse, but not at nadir. AML cells that survived initial chemotherapy exhibited a senescence-like resilient phenotype, with the potential to repopulate leukemia, regardless of whether they were LSCs or not. Upon recovery and relapse, these cells exhibited positive enrichment of LSCs signatures. Fundamental to understanding this process is the plasticity of cells, which allows them to reprogram from one metabolic profile to another. Importantly, CSCs functionality is not a fixed property but can be induced under stress conditions (Naldini et al. 2023). Some bulk RNA-seq studies also indicate that chemoresistance is driven by a senescence-like state rather than by quiescent LSCs (Farge et al. 2017; Li et al. 2023). These opposite conclusions may partially arise from the high heterogeneity of the LSCs. From a clinical point of view, a senescence-like state may explain the limited efficacy of intensified treatment regimens (Naldini et al. 2023).

### Reprogrammed metabolism: from glycolysis to lipogenesis

Glycolysis represents a central metabolic pathway crucial for stem cells differentiation. Cancer cells preferentially choose the relatively inefficient aerobic glycolysis pathway (Warburg effect) over the energy-efficient OXPHOS (oxidative phosphorylation) (Intlekofer and Finley 2019). The evidence regarding LSCs and their dependence on metabolic pathways for energy production is still conflicting. ENO1 (enolase 1), a key enzyme in the glycolytic pathway, is typically overexpressed in cancer cells. Tian, Guo, Mao et al. described one LSCs cluster characterized by the high expression of *ENO1. ENO1* knockdown promoted LSCs (HSC/MPP**-**malignant, MPP-multipotent progenitors) differentiation while inhibiting their proliferation, suggesting that ENO1 helps maintain LSCs in a quiescent state (Tian et al. 2024). A meta-analysis including 1419 AML patients, revealed that high ENO1 expression predicts poor OS (Lincz et al. 2024). Other data showed that during progression, LSCs maintain low glycolytic activity and low levels of reactive oxygen species (ROS), along with reduced expression of ENO1, ALDOA, GAPDH, and TPI1 (Stetson et al. 2021; Velasco-Hernandez et al. 2024). ). In contrast, other studies reported that AML-resistant cells are enriched for OXPHOS to meet the increased energy demands of their rapid growth (Jia et al. 2022; Chen et al. 2024). Taken together, these findings are inconclusive, highlighting the need for further investigation to clarify the metabolic profile of LSCs.

Glycosylation is a post-translational modification that adds sugar molecules to proteins or lipids, affecting their function and interaction (Nagel and Ball 2014). Hexosamine biosynthetic pathway (HBP) is a branch of glycolysis that produces UDP-GlcNAc, which is a substrate for glycosylation. The study showed that LSCs (malignant HSC/Prog-like cells according to van Galen et al. algorithm) have higher HBP activity and glycosylation rate than HSPCs, which could potentially suggest LSCs maintain low glycolysis rates by diverting more glucose to the HBP. LSCs rely on amino acid metabolism, especially glutamine, more than HSPCs to maintain OXPHOS and ensure survival. Both glucose and glutamine metabolism may regulate the HBP and O-GlcNAcylation in LSCs (Schauner et al. 2024).

During disease progression the transcriptome analysis of LSCs revealed decrease in the expression of genes involved in fatty acid synthesis, accompanied by an upregulation in mitochondrial metabolism, fatty acid oxidation, and amino acid metabolism. Genes like *CPT1C*, and *CPT2* associated with fatty acid oxidation and fatty acid transporter *CD36* were upregulated (Stetson et al. 2021). On the contrary, another study presented a noteworthy enrichment in cholesterol homeostasis in LSCs (defined above) of non-CR patients (Chen et al. 2024). Two of the four genes associated with the 4-PI score—*SQLE* and *DHCR7*—are involved in the cholesterol biosynthesis pathway, and a high 4-PI score is linked to poor prognosis. Inhibition of cholesterol synthesis enhances the anti-leukemic effects of Ara-C by decreasing the production of extracellular vesicles that deliver anti-apoptotic proteins to recipient cells- a phenomenon associated with reduced sensitivity to the Ara-C (Ortiz Rojas et al. 2024). Another study presented that quiescent-like LSCs cluster had more activated fatty acid metabolism pathways like biosynthesis, elongation, and degradation compared to proliferative LSCs clusters (Li et al. 2023). The RNA-seq revealed a novel cancer-associated gene *ARMH1* (Armadillo Like Helical Domain Containing 1). Analysis of knockdown and overexpression of *ARMH1* established an association with mitochondrial fatty acid synthesis and cell cycle pathways. *ARMH1* expression rises with the LSC6 score, reaching its maximum in AML cells with the highest score (Bakhtiari et al. 2024).

Collectively, the reported differences in metabolic dependencies likely stem from the intrinsic metabolic plasticity of LSCs, enabling them to shift between glycolysis, OXPHOS, and lipid metabolism in response to microenvironmental signals.

### Comparative analysis of matched diagnostic and post-treatment samples

Single-cell analyses typically focus on comparing matched samples collected at diagnosis and relapse or on contrasting CR and non-CR patient groups. Li et al. showed that cellular responses to chemotherapy are transient and change dynamically during cytoreduction and regeneration, accompanied by a decrease in LSCs signatures. These chemotherapy-induced dynamics, along with the inherent heterogeneity of LSCs, can lead to conflicting conclusions. A potential solution to this challenge is longitudinal single-cell analysis, which enables the tracking of dynamic cellular reprogramming across various LSCs subpopulations throughout treatment (Li et al. 2023). In the experiment performed by (Stetson et al. 2021) disease progression was largely characterized by the post-treatment loss of the cluster group that was dominant at diagnosis, along with the expansion of a relapse-dominant cluster. This finding was confirmed in another study in which the post-therapy cells were a small population of residual leukemia cells that had survived chemotherapy (Zhang et al. 2023). Most data indicate that a higher proportion of primitive or progenitor cells at relapse is associated with treatment resistance and poor prognosis (van Galen et al. 2019a; Tian et al. 2024). This applies irrespective of whether the initial tumors consisted predominantly of more differentiated or more primitive cells (Lambo et al. 2023). Using GSVA, a bioinformatics method that evaluates pathway or gene set enrichment rather than individual gene expression; Chen et al. observed that non-CR patients exhibited higher GSVA scores among LSCs compared to CR patients. Chemoresistant blasts were enriched for *HLADRB5*, *HPGD*, *S100A9*, *CCL3L1*, and *CCL4L2*. In contrast, the CR group showed enrichment of *CLEC11A*, *CFD*, *S100A10*, *LYZ*, *AZU1*, *CRIP1*, *TRH*,* MPO*, and *EGR1*. The association of *MPO* (myeloperoxidase – a hallmark lysosomal enzyme of the myeloid lineage, primarily found in neutrophils and monocytes), *TRH* (thyrotropin-releasing hormone), and *EGR1* (Early Growth Response 1) with extended RFS was confirmed by validating the findings in publicly available datasets, including TCVGH, BeatAML2, and TCGA-AML. Notably, *TRH* expression was nearly absent in LSCs from the non-CR group. In follow-up experiments, *MPO* and *TRH*—but not *EGR1*—were further validated as potential biomarkers within LSCs associated with improved survival (Chen et al. 2024). Using differentially expressed genes (DEGs), 117 genes unique to patients with a resistant phenotype were identified by Zhang et el. These genes are involved in repression of proliferation (*CDK6*, *CCND1*, *JUNB*, *SPARC*), regulation of cellular movement (*CD69*, *DUSP1*, *LGALS1*, *ANXA1*), and control of differentiation (GATA1, *CEBPA*, *RUNX1*, *ZFP36*). Additionally, genes related to the activation of OXPHOS (*SOD2*, *MT-CO2*), response to reactive oxygen species (*PRDX2*, *BTK*, *NRIP1*), and heme metabolism signaling pathways (*HBB*, *HBA1*, *HBA2*) were also enriched. Altogether, these results indicate that surviving cells acquire enhanced metabolic features while maintaining the original LSCs signatures. At the same time, all diagnostic HSPC-like populations lacking those signatures were eliminated after chemotherapy (Zhang et al. 2023).

### Strategies to target LSCs

To improve treatment outcomes in AML, it appears crucial to identify therapeutic strategies that target LSCs while sparing healthy HSCs (Ruvolo et al. 2019; Velten et al. 2021). The development of scRNA-seq has enabled the identification of novel immunotherapeutic targets and the design of personalized treatment strategies. VEN a potent BCL-2 inhibitor, combined with AZA has become the standard of care for unfit AML patients and is being evaluated in the first-line setting for fit patients. Therefore, identifying biomarkers predicting primary resistance or relapse is critical. Early predictors of VEN sensitivity included myelomonocytic and monocytic differentiation and high expression of CD11b, CD64, or CD68. The observed overexpression of BCL-2 in LSCs supports them as a key therapeutic target (Waclawiczek et al. 2023). VEN-AZA-sensitive LSCs are eliminated through inhibition OXPHOS, which is associated with mitochondrial dysfunction, SERCA (Sarco/endoplasmic reticulum Ca2+-ATPases) inhibition, and mitochondrial calcium overload (Sheth et al. 2024). In contrast, VEN-resistant LSCs maintain OXPHOS by increasing fatty acid oxidation, thereby compensating for amino acid depletion. Consequently, effective eradication of resistant LSCs requires simultaneous targeting of both amino acid metabolism and fatty acid uptake and oxidation (Cl et al. 2018). Studies are ongoing to enhance the efficacy of VEN–AZA Emerging agents such as ONC213 (Carter et al. 2025) or SLC-391 (Niu et al. 2021) Primary resistance to VEN may be driven by specific ratios of BCL-2 family proteins, representing a dominant, metabolism-independent mechanism. Accordingly, the MAC score, based on protein expression levels of BCL-2, BCL-xL, and MCL-1 specifically in LSC-like cells, predicts treatment response (Waclawiczek et al. 2023). Several other metabolic pathways and molecular targets have been identified as potential intervention points in LSCs. SMO inhibitors of the hedgehog signaling pathway, e.g. glasdegib (Tesanovic et al. 2022), HIF-inhibiting drugs (Velasco-Hernandez et al. 2024), ENO1 inhibitors (Tian et al. 2024), monoclonal antibodies targeting TIM3 like sabatolimab (Bordeleau et al. 2024). Other propositions include anti-IL2RA therapy (Ortiz Rojas et al. 2024), a selective SQLE inhibitor terbinafine (Ortiz Rojas et al. 2024), inhibition of miR-126 (Naldini et al. 2023), or transcription factors like FOS (Velten et al. 2021). Chimeric antigen receptor (CAR) T cells targeting antigens such as CD33, CD123, CLL-1 and FLT3 are promising. However, evidence of sustained complete responses in patients treated with CAR T cells has been lacking (Gomes-Silva et al. 2019). On the other hand, CAR-natural killer (NK) cells have emerged as a promising alternative to CAR-T cells due to their intrinsic potential as off-the-shelf products and safer clinical profiles e.g. TIM3 CAR-NK cells (Klaihmon et al. 2024). On overview of most advances clinical trials involving CAR-T and CAR-NK cells therapy in AML potentially targeting LSCs was shown in Table 3.

**Table 3:** Overview of most advances clinical trials involving CAR-T and CAR-NK therapy in AML potentially targeting LSCs. CAR-T cells- chimeric antigen receptor- T cells, CAR-NK cells- chimeric antigen receptor natural killer cells, AML-acute myelid leukemia, alloHSCT- allogeneic hematopoietic stem cells transplantation

Product	Target	Patient population	Clinical trial phase	ClinicalTrials.gov Identifier
VCAR33 donor-derived CD33 CAR-T cells	CD33	Relapsed/ refractory AML Adult population after alloHSCT	Phase 2	NCT05984199
CD33 CAR-T cells	CD33	Relapsed/ refractory AML Adult population	Phase 2	NCT04835519
Autologous and allogeneic CD33 CAR-T cells	CD33	Relapsed/ refractory AML Pediatric and young adult populations	Phase 2	NCT03971799
CD123 CAR-T cells	CD123	Relapsed/ refractory AML Pediatric and adult populations	Phase 2	NCT04265963 NCT04272125
ARD103 autologous CLL-1 CAR-T cells	CLL-1	Relapsed/ refractory AML Adult population	Phase 2	NCT06680752
ADGRE2 CAR-T	ADGRE2	Relapsed/ refractory AML Pediatric and adult populations	Phase 1	NCT05463640
4SCAR7U CD7 CAR-T	CD7	CD7-positive hematological malignancies Pediatric and adult populations	Phase 1	NCT05995028
CD7 CAR-T cells	CD7	Refractory/ relapsed CD7 + acute leukemias Pediatric and adult populations	Phase 2	NCT04762485
Autologous or donor-derived CD7 CAR-T cells	CD7	CD7 + hematological malignancies Pediatric and adult populations	Phase 2	NCT04033302
CD38 CAR-T cells	CD38	Relapsed/ refractory AML Pediatric and adult populations	Phase 2	NCT04351022
CD19 CAR-T cells	CD19	Relapsed/ refractory AML with t(8:21) Adult population	Phase 3	NCT04257175
TAA05 FLT3 CAR-T	FLT-3	Relapsed/ refractory AML Pediatric and adult populations	Phase 2	NCT05023707
Siglec-6 CAR-T	Siglec-6	Relapsed/ refractory AML Adult population	Phase 2	NCT05488132
EB-BH2025 Dual CD123/CD33 CAR-T cells	CD123/CD33	Relapsed/ refractory AML Pediatric and adult populations	Phase 2	NCT06420063
Dual Tim-3/ CD123 CAR-T cells	TIM-3/CD123	Relapsed/ refractory AML Adult population	Phase 2	NCT06125652
STPHI_0001 Dual CLL1/CD123 CAR-T cells	CLL1/CD123	Relapsed/ refractory AML Pediatric and adult populations	Phase 3	NCT03631576
U32 Dual CLL-1/CD38 CAR-T	CLL-1/CD38	Relapsed/ refractory AML Pediatric and adult populations	Phase 2	NCT07036250
Dual CD33-CLL1 CAR-T cells	CD33/CLL-1	Relapsed/ refractory AML Pediatric and adult populations	Phase 1	NCT05467254
Multi-CAR T Cells Muc1/CLL1/CD33/CD38/CD56/CD123	Muc1/CLL1/ CD33/CD38/ CD56/CD123	Relapsed/ refractory AML Pediatric and adult populations	Phase 2	NCT03222674
Multiple CAR-T CLL-1, CD33 and/or CD123-specific CAR gene-engineered T cells	CLL-1 and CD33/CD123	Relapsed/ refractory AML Pediatric and adult populations	Phase 2	NCT04010877
CD123 CAR-NK cells	CD123	Relapsed/ refractory AML or Blastic Plasmacytoid Dendritic Cell Neoplasm Adult population	Phase 2	NCT06006403
CD33 CAR-NK cells	CD33	Relapsed/refractory AML Pediatric and adult populations up to 39 years old	Phase 1	NCT07026942
CD33 CAR-NK cells	CD33	Relapsed/ refractory AML Adult population	Phase 1	NCT05008575
CD33 CAR-NK cells	CD33	Relapsed/ refractory AML or AML with Minimal Residual Disease	Phase 1	NCT05987696
CLL-1 CAR-NK cells	CLL-1	Relapsed/ refractory AML Adult population	Phase 1	NCT06307054 NCT06027853
CD33/CLL1 CAR-NK Cells	CD33/ CLL-1	Relapsed/ refractory AML Adult population	Early Phase 1	NCT05215015
CD7 CAR-NK cells	CD7	CD7 + relapsed/ refractory Leukemias and Lymphomas Adult population	Phase 2	NCT02742727

## Limitations

Several challenges currently limit the contribution of single-cell approaches to advancing LSCs biology. In many studies, LSCs are not the primary subject of investigation but are instead analyzed as part of secondary analyses within broader datasets, often resulting in insufficient resolution and characterization. Moreover, the lack of a standardized definition of LSCs, with different studies relying on variable phenotypic and transcriptional criteria, complicates cross-study comparisons and reproducibility. In addition, datasets are generated from heterogeneous sources, including both established cell lines and primary patient samples, which may not fully recapitulate the complexity of the disease. Collectively, these limitations introduce significant variability and impede the integration of findings, thereby limiting the interpretability and translational impact of scRNA-seq in LSCs research. Furthermore, the molecular interactions between chemoresistant LSCs and the microenvironment remain largely understudied, which could potentially be addressed through the application of spatial transcriptomics.

## Conclusions

Single-cell RNA sequencing has revolutionized the study of LSCs in AML, providing valuable insights into cellular heterogeneity and the mechanisms underlying therapy resistance. However, these studies are still at a very early stage. In the clinical context, a better understanding of metabolic pathways and surface antigen expression could inform the design of novel drugs targeting innovative mechanisms. Furthermore, improving our understanding of how transcriptomic profiles translate into protein expression will facilitate the application of this knowledge in diagnostics, in assessing treatment efficacy, and in predicting relapse. Rigorous replication of single-cell experiments remains essential to validate emerging data and distinguish true biological signals from technical artifacts. Longitudinal analyses of LSCs at clinically relevant time points—such as treatment-naïve diagnosis, chemotherapy-induced nadir, remission, and full relapse—will be essential to validate findings and capture temporal dynamics.

## Supplementary Information

Below is the link to the electronic supplementary material.

Supplementary Material 1

## Data Availability

No datasets were generated or analysed during the current study.
