# Supplementary material for: Diving into the transcriptional landscape of leukemic stem cells in acute myeloid leukemia at single-cell resolution
Source: J Cancer Res Clin Oncol. 2026 May 29;152(7):151. doi: 10.1007/s00432-026-06500-1 (PMC13421705; doi:10.1007/s00432-026-06500-1)
Supplement: Supplementary file 1 — Supplementary Material 1 [file 432_2026_6500_MOESM1_ESM.docx]

| Search number | Item | Search String |
| --- | --- | --- |
| 1 | Malignancy | “Leukemia, Myeloid, Acute" [MESH] OR "acute myeloid leukemia" OR "Acute Myelogenous Leukemia" OR "Acute Myeloblastic Leukemia" OR "Acute Myelocytic Leukemia" OR "AML" |
| 2 | Targeted cells | " Leukemic stem cells" OR "Leukemia-initiating cells" OR "LSCs" OR "LICs" OR “Cancer stem cells in leukemia” OR “Leukemia progenitor cells” |
| 3 | Method | "Single-cell RNA sequencing" OR "RNAseq" OR "Single-cell transcriptomics" OR "Single-cell RNA-seq" OR “Single-cell sequencing of RNA “ OR “scRNA sequencing” OR “Single-cell gene expression profiling” OR “Single-cell RNA profiling” OR “Single-cell transcriptome analysis” |
| 4 | Combine the above searches | 1 AND 2 AND 3 |

**Online Resource 1** Overview of PubMed search string.


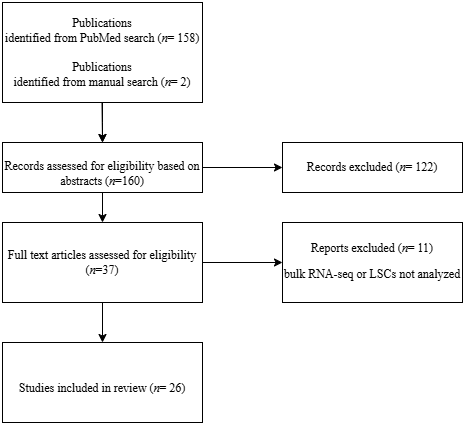


**Online Resource 2** Flow diagram summarizing the study selection process.
